# Synergistic Effect of Eugenol and Probiotic *Lactobacillus Plantarum* Zs2058 against *Salmonella* Infection in C57bl/6 Mice

**DOI:** 10.3390/nu12061611

**Published:** 2020-05-30

**Authors:** Fanfen Song, Junsheng Liu, Wenyu Zhao, Hongxuan Huang, Diangeng Hu, Haiqin Chen, Hao Zhang, Wei Chen, Zhennan Gu

**Affiliations:** 1 State Key Laboratory of Food Science and Technology, Jiangnan University, Wuxi 214122, Jiangsu, China; 17851300257@163.com (F.S.); ljshmail@163.com (J.L.); zwyjiangnan@163.com (W.Z.); hhxhuang@hotmail.com (H.H.); 7160112050@vip.jiangnan.edu.cn (D.H.); haiqinchen@jiangnan.edu.cn (H.C.); zhanghao@jiangnan.edu.cn (H.Z.); weichen@jiangnan.edu.cn (W.C.); 2 School of Food Science and Technology, Jiangnan University, Wuxi 214122, Jiangsu, China; 3 National Engineering Research Center for Functional Food, Jiangnan University, Wuxi 214122, Jiangsu, China; 4 Beijing Innovation Centre of Food Nutrition and Human Health, Beijing Technology and Business University (BTBU), Beijing 100048, China

**Keywords:** *Salmonella* infection, *Lactobacillus plantarum* ZS2058, eugenol, synergistic effect, virulence

## Abstract

Previously, we showed the preventive effects of *Lactobacillus plantarum* ZS2058 (ZS2058) on *Salmonella* infection in murine models. In this work, we found that eugenol has a selective antibacterial effect, which inhibited *Salmonella* more than probiotics ZS2058 in vitro. It suggested a synergistic effect of them beyond their individual anti-*Salmonella* activity. We verified the conjecture in murine models. The results showed that the combination of ZS2058 and eugenol (CLPZE) significantly increased (*p* = 0.026) the survival rate of *Salmonella*-infected mice from 60% to 80% and the effect of CLPZE on preventing *Salmonella*-infection was 2-fold that of ZS2058 alone and 6-fold that of eugenol alone. CLPZE had a synergistic effect on inhibiting ST growth (the coefficient drug interaction ((CDI) = 0.829), reducing its invasiveness (CDI = 0.373) and downregulating virulence genes’ expression in vitro. CLPZE helped the host form a healthier gut ecosystem. CLPZE also elicited a stronger and earlier immune response to systemic infection. In conclusion, these obtained results suggest that ZS2058 and eugenol have a synergistic effect on preventing *Salmonella* infection and open new perspectives in the strategies of controlling the prevalence of *Salmonella* by combination of probiotics and functional food components.

## 1. Introduction

Recent studies estimate that there are approximately 10–20 million cases of typhoid per year, resulting in 100,000–200,000 deaths [1,2]. After entering the gut following ingestion, *Salmonella enterica* serovar Typhi (*S*. Typhi) penetrates the intestinal epithelium and disseminates to systemic sites, including the liver, spleen, bone marrow, and gallbladder. The infectious dose of *S.* Typhi in volunteers varies between 1000 and 1 million organisms. Symptoms of typhoid typically develop 10–14 days post-ingestion, and include fever, headache, muscle aches, stomach pain, and constipation or diarrhoea [3]. Following recovery from acute disease, approximately 3–5% of infected individuals will continue to shed *S.* Typhi for several months to years [4]. As typhoidal serovars are human-restricted, carriers represent a key reservoir of *S.* Typhi, which contributes to the transmission and dissemination of typhoid [5,6]. The initial site of *Salmonella* invasion is the Peyer’s patches on the mucosal surface of the intestine, and it also colonises sites in the distal intestine, such as the cecum and colon [7]. *Salmonella enterica* serovar Typhimurium relies on two pathogenicity-islands (SPIs) encoded by type III secretion systems (T3SS), the SPI-1 and SPI-2 T3SS, for invasion and intracellular replication [8]. As *Salmonella* invades host cells, it causes inflammation and the destruction of the bowel tight junctions [9]. Antibiotics have been widely used clinically for salmonellosis. However, antibiotic treatment creates *Salmonella* persisters, which can undermine host immune defences [10]. For these reasons, it is imperative to develop novel and secure procedures for salmonellosis prevention and treatment.

Probiotics have been identified as a promising elective treatment alternative for *Salmonella* infection because these items are related to fewer side effects and superior efficacy. Many studies have investigated the ability of probiotics to prevent *Salmonella* infection, as well as the mechanisms involved. For example, *Bifidobacterium thermophilum* RBL67 was reported to modulate the transcription of virulence genes, which were identified as important contributors in *Salmonella* infection [11]. This might indicate an important mechanism that could be targeted by probiotics to reduce pathogenicity and promote pathogen clearance. Our previous work demonstrated that *Lactobacillus plantarum* ZS2058 (ZS2058) exhibited strong preventive effects against *Salmonella*-induced animal death by increasing the level of propionic acid in faeces and the production of mucin 2 in the colon, which then activated the interleukin (IL)-23/IL-22 and IL-23/IL-17 pathways in a mouse model of *Salmonella* [12,13].

Plant extracts have been used as food preservatives and dietary supplements to prevent food spoilage and maintain human health since antiquity. Our previous study showed that alcohol extract of clove, which is the aromatic flower buds of a tree, *Syzygium aromaticum*, in the Myrtaceae family, exhibited broad-spectrum bacteriostatic effects, and eugenol (4-allyl-2-methoxyphenol) is the main ingredient in several medicinal and edible plant extracts, including clove [14]. Several pharmacological activities of eugenol have been reported, including anti-inflammatory, antitumor, antibacterial, antifungal, antipyretic, anaesthetic, and analgesic activities [15], and it is generally recognised as a safe and broad-spectrum bacteriostatic. The human health safety assessment report [16] from Research Institute for Fragrance Materials (RIFM) showed that eugenol does not present genotoxicity, and it shows no repeated toxicity when daily intake is lower than 300 mg/kg/day, no observed adverse effect on development, and it reproduces when daily intake is lower than 230 mg/kg/day. The no expected sensitization induction level for skin sensitization is 5900 μg/cm^3^, and the local respiratory toxicity level is 100 mg/m^3^. The antibacterial mechanism of eugenol is diverse, including degrading the bacterial cell wall, damaging the cell membrane and membrane proteins, and then infiltration by intracellular substances [17]. Eugenol also inhibits the activity of some enzymes, such as the L-asparaginase, which involves *Salmonella* virulence mechanisms [18]. Many studies have reported the ability of eugenol to inhibit pathogenic bacteria [14,18,19], including *Salmonella*, and recent studies showed that the plant-derived antimicrobials that are highly bactericidal towards enteric pathogens exert low antimicrobial effects against commensal intestinal microbiota [20,21]. Furthermore, eugenol was also reported to protect mice from *S.* Typhimurium infection by inhibiting the type III secretion system [22].

From the above, while antibiotic abuse causes many side effects, the intake of probiotics and compounds from plant extracts as dietary supplements is likely a better choice for prevention against *Salmonella* infections. We previously showed the preventive effects of ZS2058 on *Salmonella* infection. It seems that eugenol has a selective antibacterial effect, which inhibited pathogenic bacteria more than commensal intestinal microbiota. Therefore, we inferred a synergistic effect of ZS2058 and eugenol beyond their individual anti-*Salmonella* activity. In this study, we verified the conjecture by animal experiments and studied the involved mechanism in vivo and in vitro.

## 2. Materials and Methods

### 2.1. Bacteria and Cells

*Lactobacillus plantarum* ZS2058 (ZS2058) and *Salmonella* Typhimurium SL1344 (ST) were obtained from the Culture Collections of Food Microbiology (CCFM) at Jiangnan University (Wuxi, China). The cultivation of ZS2058 and ST was the same as previously reported [13]. The *Salmonella* Typhimurium SL1344 strain showed natural resistance to streptomycin [23].

The human colorectal adenocarcinoma cell line HT-29 (ATCC HTB 38) was purchased from the Cell Bank Type Culture Collection of the Chinese Academy of Sciences (Shanghai, China). Roswell Park Memorial Institute (RPMI)-1640 medium (GIBCO BRL, Grand Island, NY, USA) supplemented with 5% (v/v) foetal bovine serum (GIBCO BRL, Grand Island, NY, USA) without penicillin–streptomycin was used for HT-29 cell culturing in an atmosphere of 5% CO_2_–95% air [13].

### 2.2. Assay for Antibacterial Activity In Vitro

ZS2058 was cultured in De Man, Rogosa, Sharpe (MRS) broth, and ZS2058 was cultured in Luria-Bertani (LB) broth. ZS2058 and ST cultures were washed three times and resuspended in (phosphate buffer saline) PBS at a concentration of 2.0 × 10^9^ colony-forming units (CFU)/mL. Eugenol was diluted at a concentration of 1 mol/L by ethyl alcohol.

For growth curve, sterile 96-well microplates were used [24]. The procedure resulted in a final concentration of the bacterial inoculum of 1 × 10^6^ CFU/mL and in a gradient of two-fold dilutions of the tested product, ranging from 12.8 mmol/L to 0.0125 mmol/L. The microplates were incubated at 37 °C for 24 h, with 4 parallels for each group. During the first 8 h, optical density (OD)600 was measured every 1 h, and during hours 8–12, OD600 was measured every 2 h.

For antibacterial activity assay, 2 × 10^8^ CFU ZS2058 for group Z (ZS2058), 8 μL 1 mol/L eugenol for group E (eugenol), 2 × 10^8^ CFU ZS2058 and 8 μL 1mol/L eugenol for group ZE (ZS2058 and eugenol), and 8 μL PBS for group C (control) were inoculated into 5 mL co-culture medium (Brain Heart Infusion Broth, BHI) containing 100 μL ST suspension respectively, and incubated at 37 °C for 18 h. Suspensions of co-cultures were serially diluted with physiological saline solution and then plated on LB agar supplemented with streptomycin at a concentration of 50 µg/mL. The CFU values of bacteria were determined after 24 h of incubation at 37 °C. The synergistic effect was accessed by coefficient drug interaction (CDI):

CDI = E_ZE_/(E_Z_ ◊ E_e_), (1)

where E_x_ = the ST amount in group ×/the ST amount in group C—there is a synergistic effect when CDI < 1. E is effect, ZE is group ZE, Z is group Z, and e is group E. The detailed calculation of CDI is shown in Supplementary Table S1.

### 2.3. Animal Experiments

Specific-pathogen-free (SPF) female C57BL/6J mice at the age of 6–7 weeks were purchased from the Charles River of Zhejiang. The mice were divided into groups and housed in a SPF room with temperature at 22 ± 4 °C and 40–70% humidity. All the animal studies were approved by the Ethics Committee of Jiangnan University (JN. No20180915c0800130), China. All aspects of the study were carried out in accordance with the European Community guidelines (Directive 2010/63/EU) for the care and use of experimental animals. ZS2058 were suspended and mixed with PBS at a final concentration of 5.0 × 10^9^ CFU/mL. ZS2058 were quantified by serial dilution and plate counting before harvesting. All the mice were fed a Purified Diet (complete formula feed, Shukbeita, Nanjing, China), and the proximate composition of closed formula diets is provided in Supplementary Table S2. For the eugenol-added diet, 0.3 g eugenol was added to every 1 kg of the Purified Diet equably. During the 10 days before infection, the mice in the control group (group C) and model group (group ST) were administered with 0.1 mL of PBS, the mice in group Z were administered with 0.1 mL of ZS2058 suspension, the mice in group E were fed with the eugenol-added diet and administered with 0.1 mL of PBS, and the mice in group ZE were fed with the eugenol-added diet and administered with 0.1 mL of ZS2058 suspension. Subsequently, the mice in group ST, group Z, group E, and group ZE were infected with 1.0 × 10^6^ CFU of *Salmonella* Typhimurium SL1344 (ST) [13]. Bacteria and PBS were administered by intragastric gavage once per day. The mortality rate was recorded daily for 20 days (*n* = 30/group). The survivors were euthanized in a CO_2_ euthanasia system (Gene&I, Beijing, China) 25 days after infection.

Another animal experiment was conducted the same as above. At 2 (*n* = 5/group) and 5 days (*n* = 5/group) post-infection, mice were deprived of food overnight and anesthetized with isoflurane in a dedicated chamber. Then blood serum, intestinal contents, intestine, liver, and serum were collected.

### 2.4. 16S rDNA Sequencing and Bioinformatic Analysis

The procedure for 16S rDNA Sequencing and bioinformatic analysis of intestinal microbiota was performed as previously described [25]. Briefly, microbial genome of DNA from mice cecal contents was extracted and purified by a Fast DNA Spin Kit for Faeces (MP Biomedicals, Irvine, CA, USA). Then, the V4 region of 16S rDNA was amplified by Polymerase Chain Reaction (PCR). After agarose gel electrophoresis, gel extraction with the QIA quick Gel Extraction Kit (Qiagen, Germany), quantification, and library preparation, sequencing was performed on an Illumina MiSeq with a MiSeq Reagent Kit according to the manufacturer’s specifications. Processing of sequence reads were performed with the QIIME package. Group significance analyses were performed using default parameters. The principal component analysis (PCA) of unweighted UniFrac distances and heatmap were created using the tools provided by https://www.metaboanalyst.ca/. The linear discriminant analysis effect size (LefSe) analysis (only those taxa that obtained a log linear discriminant analysis (LDA) score of >2 were ultimately considered) and a cladogram plot were done using the tools provided by http://huttenhower.sph.harvard.edu/galaxy.

### 2.5. Adhesion and Invasion Assay

HT-29 cells were prepared using a previously reported method [26]. Briefly, cells were seeded at a density of 4 × 10^5^ cells per well into six-well plates (GIBCO BRL, Grand Island, NY, USA) and cultured to confluent. The medium was removed, and the cells were washed with PBS. Meanwhile, ZS2058 and ST cultures were washed and resuspended in RPMI-1640 medium at a concentration of 1.0 × 10^9^ CFU/mL. Group Z and group ZE were treated with ZS2058 suspensions (2 mL) for 1 h. Then, ST suspension was added (100 μL) to each well. The ST added to group E and group ZE had been co-cultured with 0.4 mmol/L eugenol. After 1 h of incubation in a CO_2_ incubator, for the adhesion experiment, the cell monolayers were washed carefully with PBS five times and then lysed with 2 mL of 1% Triton X-100 for 20 min. For the invasion assay of ST, the procedures were the same except for gentamicin treatment (100 μg/mL, 30 min) prior to lysis with 1% Triton X-100. Serial dilutions were then performed, and the CFU counts of the adhered ST cells were enumerated by the agar pour plate method [13], with 3 parallels for each group. The calculation method of CDI is the same as that provided in Assay for Antibacterial Activity in vitro, Section 2.2. The detailed calculation of coefficient drug interaction (CDI) is show in Supplementary Table S3.

### 2.6. Determination of Virulence Genes

HT-29 cells, ZS2058, and ST suspensions were prepared as described in the Adhesion and Invasion assay Section above. Group Z and group ZE were treated with ZS2058 suspensions (2 mL) for 1 h. Then, ST suspension was added (100 μL) to each well. The ST added to group E and group ZE had been co-cultured with 0.4 mmol/L eugenol. The cells were infected with ST for 1 h, with 3 parallels for each group. Total RNA was isolated from resuspension using TRIzol (Invitrogen, Carlsbad, CA, USA). 1 μg total RNA was used to generate complementary DNA (cDNA) with the Revert Aid First Strand cDNA Synthesis Kit #K1622 (Thermo Fisher Scientific, Waltham, MA, USA) based on the manufacturer’s instructions. A polymerase chain reaction for virulence genes InvA, AvrA, SsrB, HilA, and SopD was performed with the Bio-Rad S1000 PCR (Bio-Rad, Hercules, CA, USA). The PCR program was as follows: (1) 95 °C for 5 min, (2) 28 cycles of denaturation at 95 °C for 30 s, annealing at 60 °C for 30 s, and extension at 72 °C for 30 s, and (3) 72 °C for 7 min followed by cooling at 12 °C. Primers used for this experiment are shown in Supplementary Table S4.

### 2.7. Cytokines Measurements

The fresh livers were homogenized at a 1:10 (m/v) dilution in Radio Immunoprecipitation Assay (RIPA) lysis buffer (Beyotime, Nangjing, China). The levels of various cytokines’ tissue necrosis factor (TNF)-α, interleukin (IL)-1β, and IL-6 in these homogenates were then determined using Enzyme Linked Immunosorbent Assay (ELISA) kits (R&D, Minneapolis, MN, USA).

### 2.8. Statistics

Graph Prism was used to create all figures and perform statistical analyses in this manuscript, except the microbiota analysis. One-way analysis of variance (ANOVA) was used to determine significant differences of antibacterial activity, invasiveness, α-diversity of microbiota, virulence gene expression, and cytokines among groups. A log-rank (Mantel-Cox) test was performed to analyse the difference of survival rate among groups. Alpha View SA software was used to analyse PCR bands. Data are presented as mean ± standard error of mean (SEM).

## 3. Results

### 3.1. Combination of l. Plantarum ZS2058 and Eugenol (Clpze) Exerted Synergistic Effect on the Inhibition of ST Growth In Vitro

**Figure 1:**
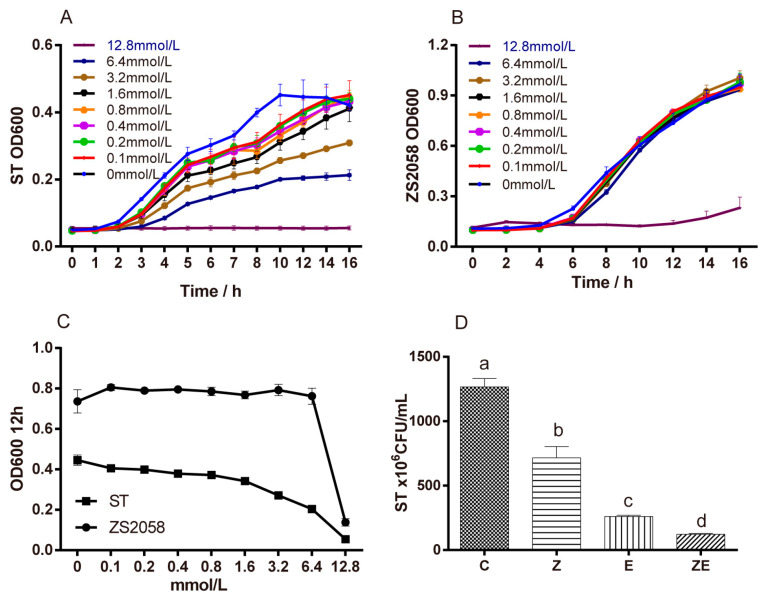
The inhibitory effects of eugenol and Lactobacillus plantarum ZS2058 on Salmonella in vitro. (**A**) Salmonella typhimurium SL1344 (ST) cultured in Luria-Bertani broth and (**B**) ZS2058 cultured in De Man, Rogosa, Sharpe (MRS) broth treated with various concentrations of eugenol. (**C**) The growth of ST and ZS2058 treated with various concentrations of eugenol at 12 h. (**D**) The antibiotic effect of eugenol and ZS2058 on ST. To compare the antibacterial effect of ZS2058 and eugenol, ST were regularly cultured (C), co-cultured with eugenol (E), co-cultured with ZS2058 (Z), and co-cultured with ZS2058 and eugenol (ZE), the CFU values of ST were counted; ST, Salmonella Typhimurium SL1344; ZS2058, Lactobacillus plantarum ZS2058. Data are expressed as means ± standard error of means (SEMs), *n* = 4. Labelled means without a common letter are significantly different, *p* < 0.05.

Firstly, we studied the sensitivity of *Lactobacillus plantarum* ZS2058 (ZS2058) and *S.* Typhimurium SL1344 (ST) to the antimicrobial activity of eugenol. The result showed that the higher the concentration of eugenol was, the stronger the inhibitory effect was on ST (Figure 1A–C). However, different concentrations of eugenol showed no significant inhibitory effects on ZS2058 when the concentration of eugenol was lower than 6.4 mmol/L. It suggests that eugenol has a stronger antibacterial effect on *Salmonella* than on ZS2058. The inhibitory effects of both *Lactobacillus plantarum* ZS2058 and eugenol on ST were also measured (Figure 1D), and both ZS2058 and eugenol had inhibitory effects on the growth of ST. When ZS2058 and eugenol were combined, the inhibitory effect was further enhanced. CLPZE had a synergistic effect on inhibiting growth of ST in vitro, as indicated by their coefficient of drug interaction (CDI = 0.827).

### 3.2. CLPZE Significantly Increased the Survival Rate of ST-Infected Mice

**Figure 2:**
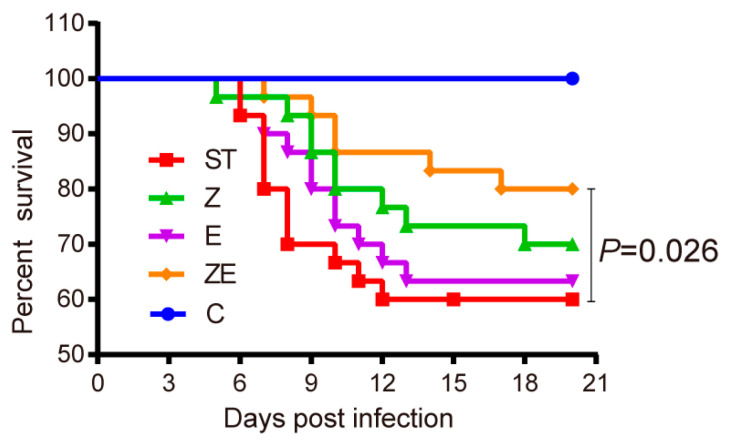
*Lactobacillus plantarum* ZS2058 (ZS2058) and eugenol muted the lethal effect of *Salmonella* in mice. Mice were pre-treated with regular diet and phosphate buffer saline (ST), regular diet and ZS2058 (Z), eugenol-contained diet and PBS (E), or eugenol-contained diet and ZS2058 (ZE) for 10 days before *Salmonella* infection. The control mice (C) were also pre-treated with regular diet and PBS but were not infected. Survival of mice was observed and recorded daily for 20 days. *n* = 30, group ZE versus group ST, *p* = 0.026; group Z versus group ST, no significance; group E versus group ST, no significance.

To further address the effects of ZS2058 and eugenol on preventing *Salmonella* infection in vivo, the mortality rate was recorded daily for 20 days post-infection. As shown in Figure 2, the survival rates of groups C, ST, Z, E, and ZE were 100%, 60%, 70%, 63.3%, and 80% respectively, at the 20th day post-infection. The survival rate of group ZE was significantly higher than group ST (*p* = 0.026). The preventive effect of CLPZE was two-fold (Equation (2)) and six-fold (Equation (3)) that of ZS2058 and eugenol alone. These data suggest the synergistic effects of ZS2058 and eugenol on preventing ST infection.

(80% − 60%)/(70% − 60%) = 2(2)

(80% − 60%)/(63.3% − 60%) = 6(3)

### 3.3. CLPZE Improves Intestinal Microbiota Structure of ST-Infected Mice

**Figure 3:**
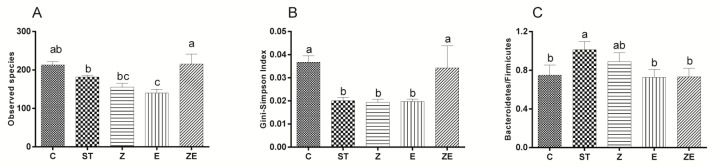
The effect of *Lactobacillus plantarum* ZS2058 (ZS2058) and eugenol on the intestinal microbiota at 2 days post-ST infection. Mice were pre-treated with regular diet and PBS (ST), regular diet and ZS2058 (Z), eugenol-contained diet and PBS (E), or eugenol-contained diet and ZS2058 (ZE) for 10 days before *Salmonella* infection. The control mice (C) were also pre-treated with regular diet and PBS but were not infected. When sacrificed at 2 days post-infection, cecal contents were collected for sequencing. (**A**) Observed species, (**B**) Gini-Simpson’s index at genus levels, and (**C**) the ratio of *Bacteroidetes/Firmicutes* of each group. Data of quantity analysis are means ± SEMs, *n* = 5. Labelled means without a common letter are significantly different, *p* < 0.05.

Alpha-diversity analysis showed that ST, ZS2058, and eugenol reduced the species richness, while CLPZE visibly maintained the richness (Figure 3A) and evenness (Figure 3B). Figure 3C shows that the *Firmicutes/Bacteroidetes* ratio, a strong indicator of gut bacterial shift, of group ST was significantly higher than other groups. Supplementation of eugenol (group E) or ZS2058 and eugenol (group ZE) prevented the ST-infection-triggered gut bacterial shift (Figure 3C).

**Figure 4:**
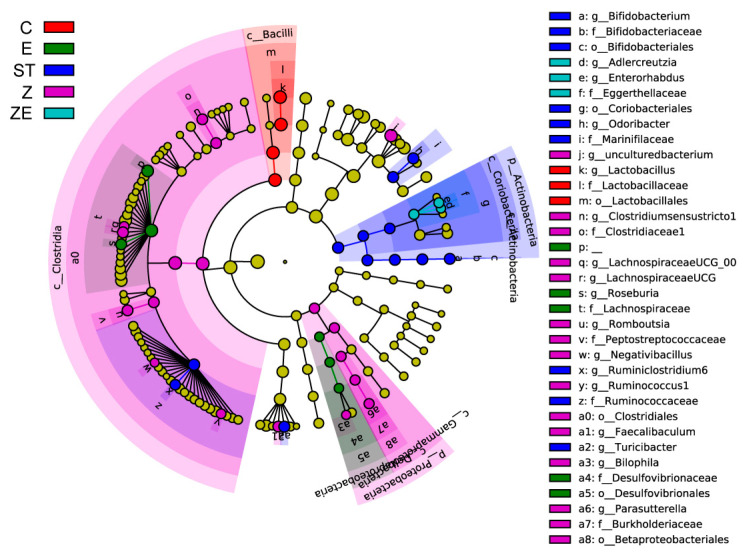
Cladogram plot showing bacterial taxa enriched in mice among groups. Mice were pre-treated with regular diet and PBS (ST), regular diet and ZS2058 (Z), eugenol-contained diet and PBS (E), or eugenol-contained diet and ZS2058 (ZE) for 10 days before *Salmonella* infection. The control mice (C) were also pre-treated with regular diet and PBS but were not infected. When sacrificed at 2 days post-infection, cecal contents were collected for sequencing. *n* = 5, only those taxa that obtained a log linear discriminant analysis (LDA) score of >2 were ultimately considered.

**Figure 5:**
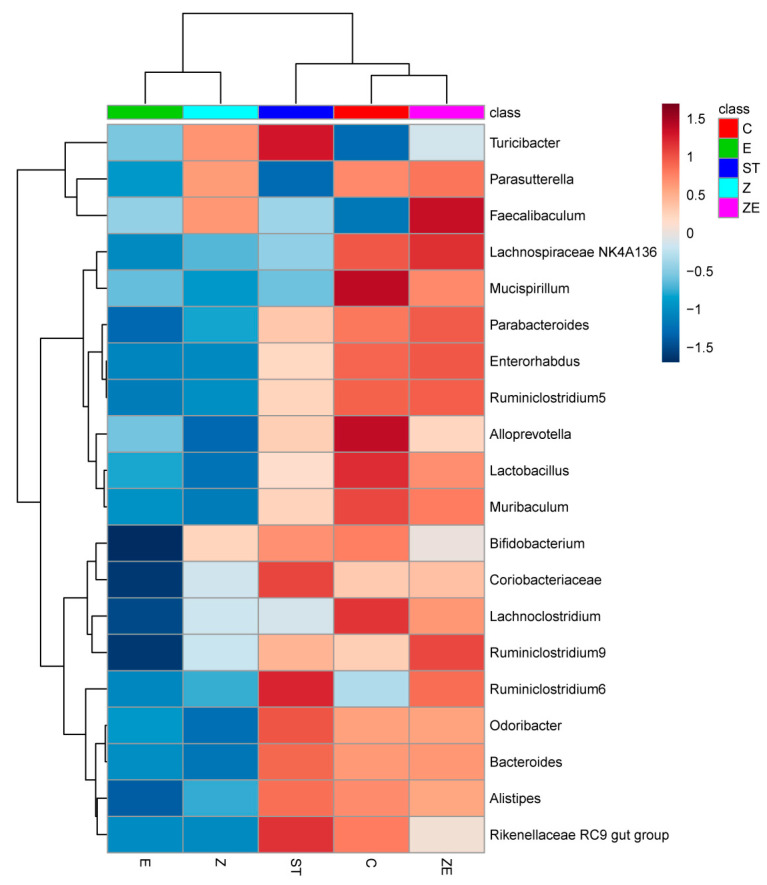
Heatmap showing the abundance difference of the gut microbiota at the genus level among groups (top 25). Mice were pre-treated with regular diet and PBS (ST), regular diet and ZS2058 (Z), eugenol-contained diet and PBS (E), or eugenol-contained diet and ZS2058 (ZE) for 10 days before *Salmonella* infection. The control mice (C) were also pre-treated with regular diet and PBS but were not infected. When sacrificed at 2 days post-infection, cecal contents were collected for sequencing. *n* = 5. Red, high abundance; white, medium abundance; blue, low abundance.

The differentially abundant bacterial taxa among these groups was exhibited in a cladogram (Figure 4) by LefSe analysis. The taxa where the top 25 members differ among groups are exhibited in the heatmap (Figure 5). Integrating the two analysis methods, the well-known probiotics *Lactobacillus* and *Mucispirillum* remained in higher abundance in group ZE and group C compared with group ST, group Z, and group E. The abundance of *Faecalibaculum* in group Z and group ZE was higher than other groups. Eugenol and ZS2058 downregulated the abundance of *Turicibacter*, which was significantly upregulated by ST infection (group ST). These results suggest that CLPZE could help to maintain or rebuild a healthier intestinal microbiota.

**Figure 6:**
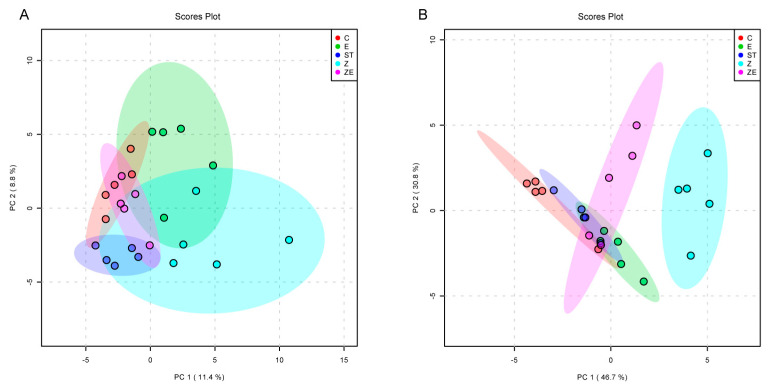
The effect of *Lactobacillus plantarum* ZS2058 (ZS2058) and eugenol on the intestinal microbiota composition at 2 days post-ST infection. Mice were pre-treated with regular diet and PBS (ST), regular diet and ZS2058 (Z), eugenol-contained diet and PBS (E), or eugenol-contained diet and ZS2058 (ZE) for 10 days before *Salmonella* infection. The control mice (C) were also pre-treated with regular diet and PBS but were not infected. When sacrificed at 2 days post-infection, cecal contents were collected for sequencing. (**A**) Principal component analysis (PCA) plot based on unweighted UniFrac metrics showing the intestinal microbiota structure of each group. (**B**) PCA plot based on unweighted UniFrac metrics showing the constituents of *Lactobacillus* genus in each group. *n* = 5.

Then the intestinal microbiota composition was compared among groups. The PCA plot (Figure 6A) revealed that the intestinal microbiota composition of group ZE was the closest to that of group C, and was clearly different to that of both group Z and group E. The composition of the *Lactobacillus* genus, to which ZS2058 belongs, was the most abundant among the detected genera (16.4% of the total), and in group Z shows the longest distance to the other groups (Figure 6B). These findings suggest that enteric dysbacteriosis happened when the mice were infected with ST, and that CLPZE contributed to a healthier gut microbial community structure and alleviated the dysbacteriosis, while neither ZS2058 nor eugenol alone could do so.

### 3.4. CLPZE Reduces Salmonella Invasiveness by Inhibiting its Virulence

**Figure 7:**
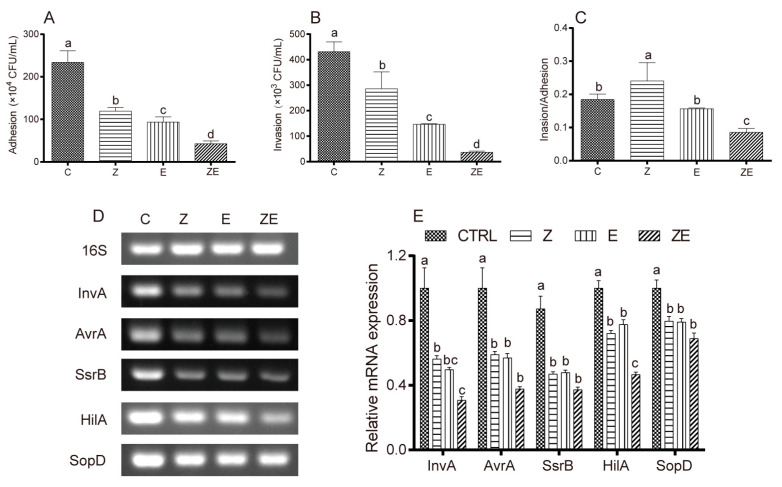
The inhibitory effects of eugenol and *Lactobacillus plantarum* ZS2058 (ZS2058) on *Salmonella* invasiveness and virulence gene expression in an HT-29 cell model. Effects of ZS2058 and eugenol on adhesion ability (**A**), invasion ability (**B**) of ST, and the invasion/adhesion ratio (**C**) in the HT-29 cell model. (**D**) The expression of the virulence genes InvA, AvrA, SsrB, HilA, and SopD by Polymerase Chain Reaction. (**E**) Grayscale analysis statistics of the electrophoretogram. To evaluate the inhibitory effect of ZS2058 and eugenol on the virulence of ST, the HT-29 cells were co-cultured with regular ST (C), eugenol-pre-treated ST (E), ZS2058 and regular ST (Z), and ZS2058 and eugenol-pre-treated ST (ZE); ST, *Salmonella* Typhimurium SL1344; ZS2058, *Lactobacillus plantarum* ZS2058. Data are expressed as means ± SEMs, *n* = 3. Labelled means without a common letter are significantly different, *p* < 0.05.

We examined the effects of eugenol and ZS2058 on the invasiveness and the virulence gene expression of ST. Both eugenol and ZS2058 reduced the adhesion (Figure 7A) and invasion (Figure 7B) of ST in an HT-29 cell model. There was a synergistic effect when eugenol and ZS2058 were combined (CDI = 0.382). Comparing the ratio of invasion/adhesion (Figure 7C), we found that CLPZE exerted stronger inhibitory effects on ST invasion than on cell adhesion.

The expression of virulence genes was inhibited to different degrees, as shown in Figure 7D,E. Compared with the control group, both ZS2058 and eugenol significantly inhibited the expression of ST virulence genes InvA, AvrA, SsrB, HilA, and SopD, and the inhibitory effect of CLPZE (group ZE) was stronger than that of either single factor alone. These results strongly suggest that CLPZE reduces the invasiveness of ST by downregulating the expression of virulence genes.

### 3.5. CLPZE Elicited Earlier Immune Responses to Prevent Systemic Infection by ST

**Figure 8:**
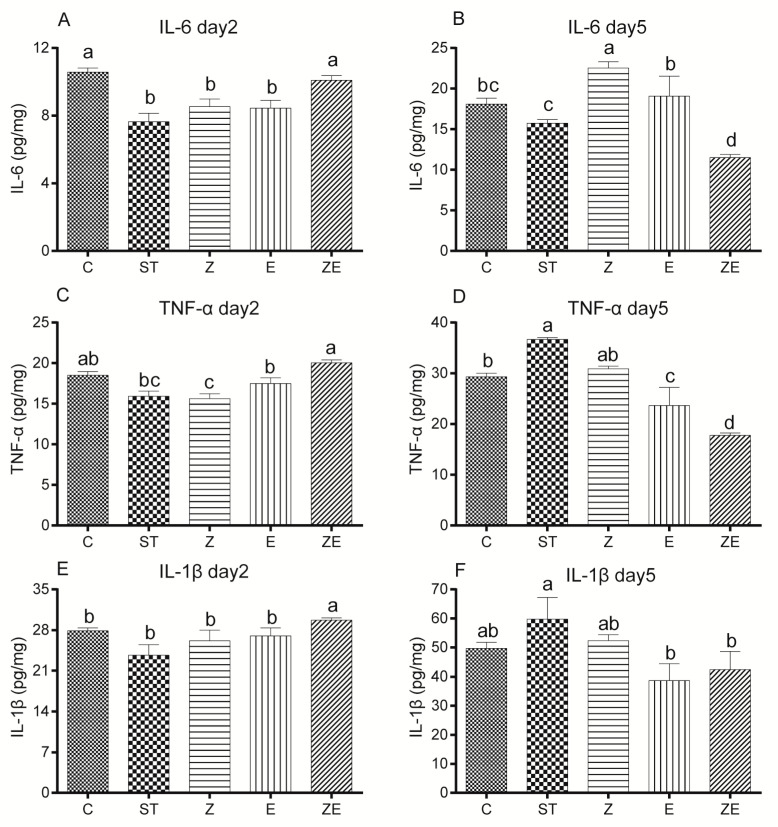
The effect of *Lactobacillus plantarum* ZS2058 (ZS2058) and eugenol on immune responses in ST-infected mice. Mice were pre-treated with regular diet and PBS (ST), regular diet and ZS2058 (Z), eugenol-contained diet and PBS (E), or eugenol-contained diet and ZS2058 (ZE) for 10 days before *Salmonella* infection. The control mice (C) were also pre-treated with regular diet and PBS but were not infected. When sacrificed at 2 (day 2) and 5 days (day 5) post-infection, liver was dissected, and the productions of cytokines detected by Enzyme Linked Immunosorbent Assay. The IL-6 level in the liver of mice at day 2 (**A**) and day 5 (**B**); the TNF-α level in the liver of mice at day 2 (**C**) and day 5 (**D**), and the IL-1β level in the liver of mice at day 2 (**E**) and day 5 (**F**). Data are expressed as means ± SEMs, *n* = 5. Labelled means without a common letter are significantly different, *p* < 0.05.

To elucidate the effect of ZS2058 and eugenol on the host immune system, we analysed the expression of pro-inflammatory cytokines IL-6, TNF-α, and IL-1β in the liver of ST-infected mice. Two days after infection (day 2), the levels of pro-inflammatory cytokines IL-6 (Figure 8A), TNF-α (Figure 8C), and IL-1β (Figure 8E) were significantly higher in the group ZE compared with the group ST. However, 5 days after infection (day 5), the levels of IL-6 (Figure 8B), TNF-α (Figure 8D), and IL-1β (Figure 8F) in group ZE were significantly lower than the model group. On the whole, the levels of pro-inflammatory cytokines in group ZE tended to coincide with group C (Figure 8), and the levels of the cytokines (Figure 8) except IL-6 (day 5) (Figure 8B) in group Z and group E were between those of group ST and group ZE. It suggests that CLPZE elicited earlier immune responses, which upregulated the inflammation at day 2 and then downregulated it at day 5, while the inflammation was significantly reduced at day 2 and raised at day 5 in the ST-infected mice.

## 4. Discussion

Our studies provide substantial evidence confirming that ZS2058 combined with eugenol (CLPZE) has a synergistic effect on preventing *Salmonella* infection. Here, we show that (1) ST showed more sensitivity to eugenol than to ZS2058, (2) CLPZE had a synergistic effect (CDI = 0.827) on inhibiting *Salmonella* Typhimurium SL1344 (ST) growth and improving survival of ST-infected mice, (3) CLPZE alleviated enteric dysbacteriosis by keeping a high diversity and healthier structure of intestinal microbiota, (4) CLPZE reduced ST invasiveness by inhibiting its virulence, and (5) CLPZE elicited earlier immune responses to prevent systemic infection by ST. In the era of worldwide concern about antimicrobial resistance, the intake of probiotics and compounds from plant extracts as dietary supplements may offer an alternative or adjunct to prevent or treat *Salmonella* infections.

While eugenol is a broad-spectrum antibacterial substance, and ZS2058 is a probiotic, we were intrigued about the effect of CLPZE on intestinal microbiota in infected mice. Highly diverse microbial communities are associated with protection against invading pathogens, and perturbations associated with reduced species richness put hosts at risk of enteric infections [27]. Diversity can be decomposed into richness and evenness [27]. In our study, ST, ZS2058, and eugenol all reduced the intestinal microbiota richness (Figure 3A) and evenness (Figure 3B), while CLPZE retained the richness and evenness close to those of the normal mice. Interestingly, CLPZE retained or upregulated the abundance of some beneficial bacterial genera, such as the well-known probiotics *Lactobacillus*, anti-inflammation and butyrate-producing *Faecalibaculum* [28], and *Mucispirillum*, a nutritional competitor of *Salmonella* [29]. *Turicibacter*, which was reported to significantly increase in mice during or after the development of colitis or colorectal cancer [30], decreased in group ZE, while it was significantly upregulated by ST in group ST. Overall, the results strongly suggest that CLPZE assists the host in retaining a healthier gut microbial environment to protect against invading pathogens.

*Lactobacillus* deserves our special attention because it is the genus to which ZS2058 belongs and the most abundant among the detected genera (16.4% of the total). *Lactobacillus* species have been well characterised for their potential resistance to the major gastric and enteric bacterial pathogens, due to their products such as short-chain fatty acids (SCFA) and lactic acid and their occupying effect on gut mucosal cells [31]. The abundance of *Lactobacillus* in group ZE showed no significant difference with the control group, while it was downregulated in ST, ZS2058, and eugenol groups (Figure 5). The lower abundance of *Lactobacillus* in group E may be due to the broad-spectrum antibacterial effect of eugenol. As for the lower abundance of *Lactobacillus* in group Z, we make the following conjecture. Our previous studies had reported that the significantly higher level of faecal SCFA was an important factor for the preventive effect of ZS2058 on salmonellosis in mice [12]. However, the level of SCFA might not be a reliable reference for the improvement of intestinal microbiota diversity by CLPZE, because the faecal SCFA content in group ZE was not increased (Supplementary Figure S1). The microbiota composition was highlighted in mediating divergent host outcomes and infection dynamics [27], and we consider the strains in the *Lactobacillus* genus as a functional group and focus on the composition of *Lactobacillus* in each group. The composition of *Lactobacillus* in group Z showed the biggest difference from group C compared with other groups (Figure 6B). It indicates that the downregulated abundance of *Lactobacillus* and intestinal microbiota diversity induced by ZS2058 might be the consequence of interspecies competition [32] and the internal disorder of the *Lactobacillus* functional group.

The adult human intestine hosts 10^13^ to 10^14^ bacteria belonging to at least 500 different species or strains [2]. Up to nine different bacterial phyla are usually found; however, the *Firmicutes* and *Bacteroidetes* account for over 90% of all bacteria [3]. It was suggested that the high susceptibility to bacterial pathogens of infants and the elderly is due to the proportion of *Bacteroidetes/Firmicutes* in the intestinal microbiota being much higher than in normal people [33]. In the current study, the *Bacteroidetes/Firmicutes* ratio of group ST was significantly higher than that of group C, group Z, and group ZE. It suggested that ST reduced host resistance to pathogens, and that ZS2058 and CLPZE restored such resistance.

The theory that CLPZE helped the host retain a healthier gut ecosystem when ST was invading was supported by the retained intestinal microbiota diversity, the abundance and evenness of *Lactobacillus*, a lower *Bacteroidetes/Firmicutes* ratio, and the retained or upregulated beneficial genera (*Faecalibaculum*, *Lactobacillus*, *Mucispirillum*). We hypothesise that when ZS2058 and eugenol were ingested, eugenol showed a stronger bactericidal effect towards enteric pathogens such as ST (Figure 1) and that it also diminished colonisation of ZS2058, which helped maintain a healthy *Lactobacillus* functional group. Therefore, the inhibitory effects on ST were increased and some beneficial genera were enriched. After all, these explanations remain to be verified.

The pathogenicity of *Salmonella* is triggered by host cell invasion, followed by intracellular survival and colonisation [34], which all rely on the virulence genes of *Salmonella*. *Salmonella* uses a multitude of virulence factors to overcome the mucosal barrier and evade the cellular and humoral host defences [35]. Our previous studies have shown that ZS2058 can reduce the adhesion and invasion ability of *Salmonella*, which is potentially ascribed to the inhibition of *Salmonella* virulence genes [13]. Significant inhibitory effects on *Salmonella* infection by altering the function of the Type III secretion system (T3SS) have been reported [22]. The virulence genes InvA, AvrA, HilA, SsrB, and SopD play vital roles in the ST T3SS. Effector molecules secreted by the SPI1-T3SS act during the early phase of infection and enable *Salmonella* to penetrate the intact intestinal epithelial barrier and reach the subepithelial tissue [36,37]. The main transcriptional activator HilA is required for a functional SPI1-T3SS. It is activated upon arrival to the distal ileum of the small intestine, allowing *Salmonella* to invade epithelial cells [38]. InvA is the key to local inflammation induction and the pathogenesis of *Salmonella* [39]. The acetyltransferase activity of AvrA can suppress inflammation triggered by apoptosis of macrophages and enhance bacterial intracellular survival [40], inhibit the secretion of cytokines IL-12, IFN-γ, and TNF-α, and enhance *Salmonella* invasiveness [41]. SsrB, a transcriptional regulator partially controlled by PhoPQ two-component regulatory system, has been shown to be a link between SPI2 and SPI1 regulatory pathways [38,42]. SsrB repressed SPI-1 by regulating HilD and HilA genes, which significantly reduced the invasion ability of *S.* Typhimurium while promoting the expression of genes required for intracellular survival [42]. SopD contributes to a variety of post-invasion processes, such as vacuolation [43] and modulation of the inflammatory response [44]. In our study, ZS2058 and eugenol had a synergistic effect on inhibiting the invasion of ST (CDI = 0.373) in a HT-29 cell model by downregulating the expression of virulence genes InvA, AvrA, HilA, SsrB, and SopD (Figure 7). The results from HT-29 cell models were comparable with in vivo observations [13]. These results indicate that CLPZE could reduce the invasiveness as well as the intracellular viability of ST in vivo.

In addition, the *Mucispirillum* genus, including the species *Mucispirillum schaedleri*, which was reported to protect mice against enteric *Salmonella* infection by competing for nitrate in the gut and interfering with pathogen invasion and virulence factor expression [29], was restored by CLPZE, while the abundance of *Mucispirillum* was significantly downregulated by ST (Figure 5), which may provide more evidence for downregulation of ST virulence genes in vivo.

After entering the guts, *S*. Typhi penetrates the intestinal epithelium and systemic infection is triggered [3]. On the one hand, the infection-activated innate immune system can eliminate pathogens [45]. On the other hand, systemic infection can result in life-threatening thrombosis which is followed by local inflammation and upregulation of podoplanin and platelet activation within the liver [46]. However, differential regulation of *Salmonella* SPI-1 restrains *S.* Typhi from triggering a pronounced, immediate inflammatory response in the host [35]. Consistent with this, ST downregulated the inflammation level in the liver at day 2 and upregulated it at day 5 (Figure 8). In contrast to group ST, the levels of pro-inflammatory cytokines IL-1β, IL-6, and TNF-α in the liver of the mice in group ZE were raised at day 2 and declined at day 5 (Figure 8). This might be due to the downregulation of ST virulence genes, especially AvrA. It is also possible that CLPZE enhances host antibacterial immune responses, as eugenol was reported to alleviate visceral leishmaniasis via immunomodulation [47]. Our previous studies also reported that ZS2058 protects ST-infected mice by activating the IL-23/IL-22 and IL-23/IL-17 pathways [12,13]. Together, these results suggest that CLPZE enables the host to detect and eliminate ST in time by activating the inflammatory response.

There are also some shortcomings in this manuscript. While CLPZE provided some beneficial intestinal microbiota changes, which have been correlated with preventing ST infection, the functional importance of these associations has not been thoroughly investigated. The antimicrobial effect of eugenol on ZS2058 in mice remains unclear, and the effects of CLPZE on ST survival virulence in vivo should be addressed. The therapeutic action of ZS2058 and eugenol also deserves further study.

## 5. Conclusions

In conclusion, our work revealed the distinct phenomenon that a combination of *Lactobacillus plantarum* ZS2058 and eugenol (CLPZE) showed a synergistic effect on preventing survival of ST-infected mice. On the one hand, CLPZE helped the host form a healthier gut ecosystem, including preserving the intestinal microbiota diversity, keeping the abundance and evenness of the *Lactobacillus* genus, downregulating the *Bacteroidetes*/*Firmicutes* ratio, and upregulating some beneficial species. On the other hand, CLPZE reduced invasiveness of ST by downregulating virulence, including InvA, AvrA, HilA, SsrB, and SopD expression, and directly inhibited the growth of *Salmonella* in vitro. These findings suggest the great potential of ZS2058 and eugenol to prevent the prevalence of this dominant pathogen.

## Supplementary Materials

The following are available online at https://www.mdpi.com/2072-6643/12/6/1611/s1: Figure S1: *Lactobacillus plantarum* ZS2058 increased the content of short chain fatty acids in mice faeces. Table S1: The coefficient drug interaction (CDI) of eugenol and *Lactobacillus plantarum* ZS2058 in inhibiting *Salmonella Typhimurium* SL1344 growth. Table S2: The proximate composition of the closed formula Purified Diet. Table S3: The drug interaction (CDI) of eugenol and *Lactobacillus plantarum* ZS2058 in inhibiting *Salmonella Typhimurium* SL1344 invasiveness. Table S4: Primers used for PCR or qPCR analysis.
