# Supplementary material for: Synergistic Effect of Eugenol and Probiotic Lactobacillus Plantarum Zs2058 against Salmonella Infection in C57bl/6 Mice
Source: Nutrients. 2020 May 30;12(6):1611. doi: 10.3390/nu12061611 (PMC7352263; doi:10.3390/nu12061611)
Supplement: Supplementary file 1 [file nutrients-12-01611-s001.pdf]

Supplementary Material

Synergistic effect of eugenol and probiotic *Lactobacillus plantarum* ZS2058 against *Salmonella* infection in C57BL/6 Mice

Fanfen Song[1,2], Junsheng Liu[1,2,3], Wenyu Zhao[1,2], Hongxuan Huang[1,2], Diangeng Hu[1,2], Haiqin Chen[1,2], Hao Zhang[1,2,3], Wei Chen[1,2,3,4], Zhennan Gu*[1,2]

[1] State Key Laboratory of Food Science and Technology, Jiangnan University, Wuxi, Jiangsu 214122, P. R. China; 17851300257@163.com (F.S.); ljshmail@163.com (J.L.); zwyjiangnan@163.com (W.Z.); hhxhuang@hotmail.com (H.H.); 7160112050@vip.jiangnan.edu.cn (D.H.); haiqinchen@jiangnan.edu.cn (H.C.); zhanghao@jiangnan.edu.cn (H.Z.); weichen@jiangnan.edu.cn (W.C.); zhennangu@jiangnan.edu.cn (Z.G.)
[2] School of Food Science and Technology, Jiangnan University, Wuxi, Jiangsu 214122, P. R. China; 17851300257@163.com (F.S.); ljshmail@163.com (J.L.); zwyjiangnan@163.com (W.Z.); hhxhuang@hotmail.com (H.H.); 7160112050@vip.jiangnan.edu.cn (D.H.); haiqinchen@jiangnan.edu.cn (H.C.); zhanghao@jiangnan.edu.cn (H.Z.); weichen@jiangnan.edu.cn (W.C.); zhennangu@jiangnan.edu.cn (Z.G.)
[3] National Engineering Research Center for Functional Food, Jiangnan University, Wuxi, Jiangsu 214122, P. R. China; ljshmail@163.com (J.L.); zhanghao@jiangnan.edu.cn (H.Z.); weichen@jiangnan.edu.cn (W.C.)
[4] Beijing Innovation Centre of Food Nutrition and Human Health, Beijing Technology and Business University (BTBU), Beijing 100048, P. R. China; weichen@jiangnan.edu.cn (W.C.)
* Correspondence: zhennangu@jiangnan.edu.cn; Tel.: +86-153-5800-0855

Received: 29 April 2020; Accepted: 27 May 2020; Published: 30 May 2020

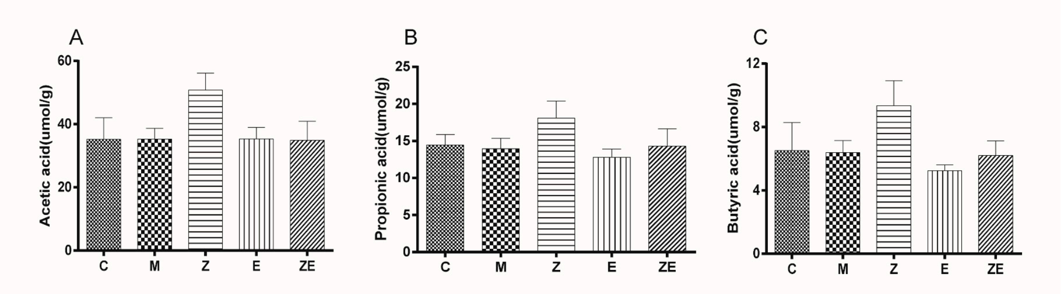

Figure S1. *Lactobacillus plantarum* ZS2058 increased the content of short chain fatty acids (SCFA) in mice feces. The level short chain fatty acids including acetic acid (**A**), propionic acid (**B**), and butyric acid(**C**) in feces of mice. Mice were pre-treated with regular diet and PBS (ST), regular diet and ZS2058 (Z), eugenol-contained diet and PBS (E), or eugenol-contained diet and ZS2058 (ZE) for 10 days. The control mice (C) were also pre-treated with regular diet and PBS but were not infected. Mice feces were then collected and Concentrations of SCFAs were measured as previously reported[1]. Briefly, feces were collected, freeze-dried and then weighed. Subsequently, the fecal matter was soaked in saturated sodium chloride and treated with aqueous sulfuric acid and diethyl ether for acidification and extraction, respectively. The SCFA analysis was performed via gas chromatography-mass spectrometry on a GCMS-QP2010 Ultra device (Shimadzu Co., Tokyo, Japan). Data are expressed as means ± SEMs, n = 5.

Table S1. The coefficient drug interaction (CDI) of eugenol and *Lactobacillus plantarum* ZS2058 in inhibiting *Salmonella Typhimurium* SL1344 growth.

Group	Parallel samples (× 10^6 CFU/mL)						Mean
C	1170	1280	1200	1300	1300	1340	1265
Z	720	750	630	840	750	600	715
E	265	250	268	267	248	268	261
ZE	130	117	119	130	115	121	122
CDI	(122/1265)/ [(715/1265) × (265/1265)] = 0.829						

To compare the antibacterial effect of ZS2058 and eugenol, ST were regularly cultured (C), co-cultured with eugenol (E), co-cultured with ZS2058 (Z), and co-cultured with ZS2058 and eugenol (ZE), the CFU values of ST were counted; ST, Salmonella Typhimurium SL1344; ZS2058, Lactobacillus plantarum ZS2058.

$$CDI = E_{ZE} / (E_Z \times E_e), \quad (1)$$

E_x = the ST amount in group x / the ST amount in group C; there is a synergistic effect when CDI < 1; E, effect; ZE, group ZE; Z, group Z; e, group E.

Table S2. The proximate composition of the closed formula Purified Diet.

main nutrients	The Purified Diet (g/kg)	Eugenol added diet (g/kg)
Water	⩽ 100	⩽ 100
Crude protein	⩾ 220	⩾ 220
Crude fat	⩾ 40	⩾ 40
Crude fibre	⩽ 50	⩽ 50
Crude ash	⩽ 80	⩽ 80
Calcium	10-18	10-18
total phosphorus	6-12	6-12
Calcium/ total phosphorus	1.2/1.7/1	1.2/1.7/1
Lysine	⩾ 13.2	⩾ 13.2
Methionine + Cystine	⩾ 7.8	⩾ 7.8
Eugenol	0	0.3

Table S3. The drug interaction (CDI) of eugenol and *Lactobacillus plantarum* ZS2058 in inhibiting *Salmonella Typhimurium* SL1344 invasiveness.

Group	Parallel samples (× 10^3 CFU/mL)						Mean
C	489	448	421	397	449	384	431.3
Z	202	356	312	205	315	326	286.0
E	143	148	140	149	146	151	146.2
ZE	34	36	38	40	28	42	36.3
CDI	(36.3/431.3)/ [(286/431.3) × (146.2/431.3)] = 0.373						

To evaluate the inhibitory effect of ZS2058 and eugenol on the virulence of ST, the HT-29 cells were co-cultured with regular ST (C), co-cultured with eugenol pretreated ST (E), co-cultured with ZS2058 and regular ST (Z), and co-cultured with ZS2058 and eugenol-pre-treated ST (ZE); ST, *Salmonella* Typhimurium SL1344; ZS2058, *Lactobacillus plantarum* ZS2058.

$$CDI = E_{ZE} / (E_Z \times E_e), \quad (1)$$

E_x = the ST amount in group x / the ST amount in group C; there is a synergistic effect when CDI < 1; E, effect; ZE, group ZE; Z, group Z; e, group E.

Table S4. Primers used for PCR or qPCR analysis.

Gene	Primers sequence（5′-3′）
InvA	F: CATTAACCTTGTGGAGCATATTCG<br>R: CATCCTCAACTTCAGCAGATACC
SopD	F: GGACGCTTCTCAGACACAAT<br>R: CGGGACGCATCATCTCATAA
AvrA	F: TGTCCAGTTACGAAGCCACAAA<br>R: TCAATACCTGCCTTTCTGTAGCC
HilA	F: CGACTCATACATTGGCGATACTT<br>R: CGGCAGTTCTTCGTAATGGT
SsrB	F: AAGGCTGTTTAGGTCAAATAGGGC<br>R: TCAAGGATAAGTATGTCAGGCTCGT
16S rRNA	F: CGATGTCTACTTGGAGGTTGTG<br>R: CTCTGGAAAGTTCTGTGGATGTC

References

1. Li, X.; Xu, Q.; Jiang, T.; Fang, S.; Wang, G.; Zhao, J.; Zhang, H.; Chen, W. A comparative study of the antidiabetic effects exerted by live and dead multi-strain probiotics in the type 2 diabetes model of mice. *Food Funct* **2016**, *7*, 4851-4860, doi:10.1039/c6fo01147k.
